# Stochastic and Statistical Analysis of Utility Revenues and Weather Data Analysis for Consumer Demand Estimation in Smart Grids

**DOI:** 10.1371/journal.pone.0156849

**Published:** 2016-06-17

**Authors:** S. M. Ali, C. A Mehmood, B. Khan, M. Jawad, U Farid, J. K. Jadoon, M. Ali, N. K. Tareen, S. Usman, M. Majid, S. M. Anwar

**Affiliations:** 1 Electrical Engineering Department, COMSATS Institute of IT, Abbottabad, KPK, Pakistan; 2 Electrical Engineering Department, COMSATS Institute of IT, Lahore, Pakistan; 3 Computer Science Department, COMSATS Institute of IT, Abbottabad, KPK, Pakistan; 4 Electrical Engineering Department, COMSATS Institute of IT, Sahiwal, Pakistan; 5 Computer Engineering Department, University of Engineering and Technology, Taxila, Pakistan; 6 Software Engineering Department, University of Engineering and Technology, Taxila, Pakistan; Chongqing University, CHINA

## Abstract

In smart grid paradigm, the consumer demands are random and time-dependent, owning towards stochastic probabilities. The stochastically varying consumer demands have put the policy makers and supplying agencies in a demanding position for optimal generation management. The utility revenue functions are highly dependent on the consumer deterministic stochastic demand models. The sudden drifts in weather parameters effects the living standards of the consumers that in turn influence the power demands. Considering above, we analyzed stochastically and statistically the effect of random consumer demands on the fixed and variable revenues of the electrical utilities. Our work presented the Multi-Variate Gaussian Distribution Function (MVGDF) probabilistic model of the utility revenues with time-dependent consumer random demands. Moreover, the Gaussian probabilities outcome of the utility revenues is based on the varying consumer *n* demands data-pattern. Furthermore, Standard Monte Carlo (SMC) simulations are performed that validated the factor of accuracy in the aforesaid probabilistic demand-revenue model. We critically analyzed the effect of weather data parameters on consumer demands using correlation and multi-linear regression schemes. The statistical analysis of consumer demands provided a relationship between dependent (demand) and independent variables (weather data) for utility load management, generation control, and network expansion.

## 1. Introduction

For evaluating probabilities of various events in Smart Grids (SGs), statistical analysis plays a fundamental role in stochastic processes [1]. Every time varying event in smart is stochastic, such as consumer demand and utility revenues. Probabilistic models of the utility revenues will estimate present and future outcomes from the random consumer demand. Random distributions, such as the Gaussian Distribution Function (GDF) will predict utility outcomes with various samples of random data. The GDF will help the policy makers to re-shape and modify the current and future utility expansion plans for modeling large scale distribution [1].

The utility costs, such as running costs, maintenance costs, operational costs, and wages and salaries of the crews have put a limit on the net utility revenues (fixed and variable) [2]. The fluctuating load curves, seasonal variations, weather drifts, and living standards of the consumers have resulted in stochastic energy demands [3],[4],[5]. Energy consumption in each inter-connected area of the SG is time-variant. Utility demand curve flattening during off-peak period, mid-peak period, and peak periods is still a big issue of the electrical utilities [6], [7]. In the aforementioned scenario, probabilistic analysis of demand-revenue model is challenging task for the optimized utility outcomes.

The stochastic load growth in the smart grid system will result in various issues, such as demand-supply miss-match, voltage instability, transmission line losses, and blackouts [8], [9].The steady-state performance, stability, and control of the SG will be affected by the above stated issues. In deregulated energy market, the revenue of the energy supplying agencies (utilities) is highly dependent on the consumer’s participation in the energy demand-response programs [10]. The reliability and quality of energy service will be degraded, resulting in less customer participation and less revenue generation [11]. Lack of past, present, and future probabilistic demand information will prevent utilities from intelligent demand-supply management and maximization of profits. The drifts in climate effects the life-styles of the consumers that influence their living standards. This variation indirectly effects the consumer demands [12], [13], [14]. Therefore, a model interpreting load relationship with weather parameters will reflect the aforesaid dependency [15].

In the light of above, there is a pressing need to develop stochastic smart grid models and statistical analysis of consumer demands. We present in depth analysis of the Multi-Variate Gaussian Distribution Function (MVGDF) model for utility revenues with stochastic consumer demands. The consumer demands are modeled as a stochastically time-dependent processes with various data samples *n*, such as *n* = 10^7^, n = 10^8^, and *n* = 10^9^. Moreover, we also elaborate random classification of the consumer demand and utility revenue for the outcomes of the MVGDF. Furthermore, the probabilistic utility revenues are estimated for present and future grid planning and management. Finally, the relative errors in the estimated models are evaluated for various data samples and statistics of the proposed models are comparatively analyzed. Our work also presents the effect of climatic change on consumer demands and penetration level of weather parameters in consumer load estimations. We believe that our research contribution is more versatile and covers a broad area in the SG stochastic processes and time-variant demand patterns, compared to prior works.

The main contributions of our paper in the light of the above stated issues are:

- We present a mathematical MVGDF model for estimating probabilities of the utility revenues with time-varying consumer demands during off-peak period, mid-peak period, and peak periods
- Our work describes a detailed statistical and comparative analysis of aforementioned models for various time-variant demand samples, such as *n* = 10^8^ and *n* = 10^9^
- The estimated values are elaborated for estimating the present and future utility revenues, which will help in various utility growth factors, such as utility planning, utility economic and financial developments, and utility network expansion
- Relative Errors (REs), Confidence Intervals (CI), and G-Matrix Scatter plots are analyzed in the aforesaid models with a brief discussion on the factor of accuracy in proposed estimations; and
- The MVGDF model is validated using Standard Monte Carlo (SMC) simulations for various consumer energy demands; and
- Correlation and regression analysis is also presented for elaborating the inter-dependency between weather data parameters and the corresponding relationship for consumer demands estimation.

The remainder of the paper is structured as follows. Section 2 discusses the related work on the stochastic processes of the SG and probability models for the utility revenues. The mathematical model of MVGDF is elaborated in Section 3. SMC Simulations and weather data analysis is described in Section 4. Section 5 concludes the paper with a summary and proposal for future work.

## 2. Related Work

One thread of the research focusses on the stochastic models and processes in the SG environment. The stochastic and prediction model for an efficient energy flow to achieve load balancing and minimize fluctuations during demand curve periods is presented in [16]. The unit commitment problem for demand-supply balance with renewable energy resources using hidden markov chains is addressed in [17]. The authors in [18] analyzed the impact of hybrid electric vehicle charging on the solar power grid inter-connected system using intelligent stochastic models. The optimized power consumption model for load scheduling using constrained Markov decision process is described in [19]. Although the aforementioned stochastic schemes presented optimized models for power consumption and demand-supply balance, they are unable to analyzed time varying consumer demands, which we proposed in our MVGDF model. Moreover, such schemes are unable to define the stochastic impact of power consumption on electrical utilities, such as revenue generation.

A large body of the research community focusses on the probabilistic load forecasting and demand-response models in SGs. The authors in [20] and [21] proposed stochastic load models using advanced energy metering and consumer’s appliances. A similar schemes presented in [22] using probabilistic demand-response for consumer’s load management. The authors in [23] formulated robust uncertainty model in SG, while generation expansion and planning schemes are proposed in [24]. The aforesaid methodologies described uncertainty in SG model and design but are not scalable enough to simultaneously predict consumer’s demand. Moreover, they suffer from comparative statistical analysis for SG modeling. Furthermore, the above schemes were unable to elaborate the impact of forecasted load on the utilities revenue.

Apart from modeling uncertainty in consumer’s load, few existing techniques were based on probabilistic models for the revenue maximization and estimation. Day-a-head pricing model for revenue maximization is described in [25], while a similar work using agent-based SG planning is presented in [26]. An optimization problem for increasing revenues from renewable energy resources with dynamic weather conditions is addressed in [27]. The authors in [28] discussed an optimal way of vehicle-to-grid charging and parking for increasing the utility revenues. The above mentioned schemes unable to evaluate the statistical analysis for probabilistic estimation. Consequently, we incorporate detailed statistical analysis and the effects of random demand inputs on the utility outcomes.

Most of the aforementioned approaches either focus on load forecasting or revenue maximization using renewable energy resources. These schemes do not thoroughly investigate and analyze the statistical behavior of consumer’s random demand on the utility revenues. Moreover, the forecasted models are unable to estimate the relative errors in their predictions and probability estimates. Furthermore, the probabilistic models are not validated using distribution density functions, such as GDF. Consequently, our work provides a thorough treatment to the problem at hand, with a complete theoretical and simulation validation.

## 3. Problem Formulation

**Fig 1:**
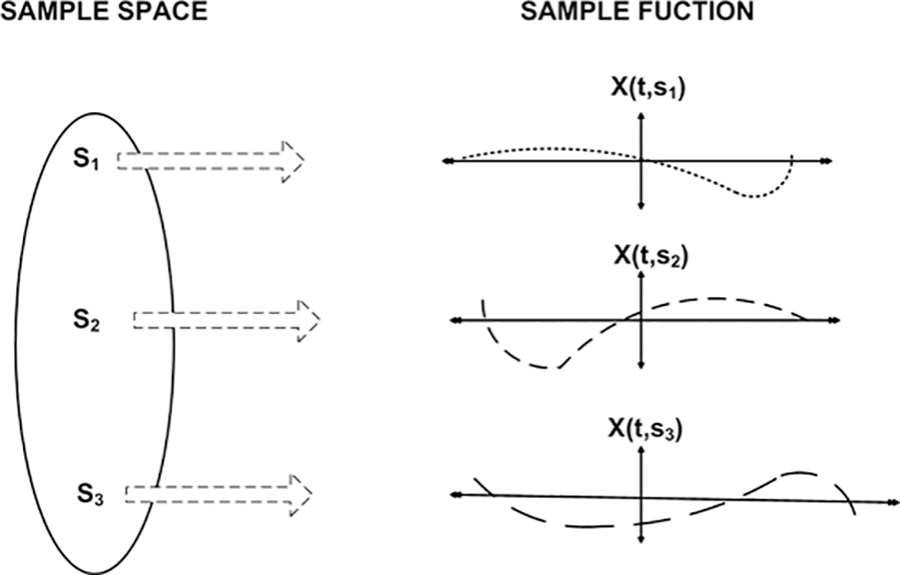
Conceptual overview of stochastic process.

In SG systems, randomly varying consumer demand is a stochastic problem for the optimal generation of the electrical utility revenues. The conceptual picture of a stochastic system is presented in Fig 1. Electrical utilities are facing a challenging problem of revenue maximization with randomly varying daily load curve, monthly load curve, and yearly load curve. The running, operational, and maintenance costs of the electrical grid with wages and salaries of electrical crews have put a limit on the resulting profit of the supplying agencies. The probabilistic revenue function is a random process dependent on the time varying load patterns. Thus, a revenue function is a time function with stochastic experiment of varying consumer demands.

The sample space is described by three random samples, namely **S**_**1**_, **S**_**2**_, and **S**_**3**_. The outcomes of the random experiment vary stochastically. The sample function of each random output is defined as: **X (t, S**_**1**_**)**, **X (t, S**_**2**_**)**, and **X (t, S**_**3**_**)**. With varying dynamics of the SG, a stochastic process will lend itself to averages estimation. The probability models with *n* number of samples will result in an optimized outcome with random and time dependent inputs. The electrical utilities can predict from the probabilistic models the present and future outcomes from time varying load patterns. Moreover, relative errors in actual and estimated values will reflect the factor of accuracy in proposed model. Furthermore, the Gaussian Probability Density Function (GPDF) with maximum data samples will produce the statistical results of the utility revenues. Finally, numerical and graphical results will provide complete analytical overview of the utilities revenues.

### 3.1 Stochastic Analysis of the MVGDF

The normal random variables are referred as Gaussian random variables. The MVGDF is a mathematical model for *n* number of variables exhibiting a Gaussian Property (GP). The GP illustrates that all the random variables possesses the probability density function to be strictly Gaussian. The set that presents all MVGDFs are jointly Gaussian. The MVGDF is presented considering the consumer demands to be strictly Gaussian. The estimated utility revenues are also Gaussian. Let $\hat{R}$ be a Gaussian (*μ*,*σ*) Random Variable (GRV) with Probability Density Function (PDF) of *X* described as:

$${\hat{R}}_{x}(x)=\frac{1}{\sqrt{2\pi \sigma^{2}}}\exp\left[\frac{-{\left(x-\mu_{x}\right)}^{2}}{2\sigma^{2}}\right],$$ (1)

The constraints of ${\hat{R}}_{x}(x)$ are defined as

1. ${\hat{R}}_{x}(x)$ is valid ∀(Re(*μ*) *and σ* > 0),
2. ${\hat{R}}_{x}(x)\approx N(\mu ,\sigma^{2}),$
3. $\int_{-\infty }^{\infty }f_{x}(x)dx=1.$

The standard normal Cumulative Density Function (CDF) of random variable *Z* is defined as:

$$\Phi (z)=\frac{1}{\sqrt{2\pi }\int_{-\infty }^{\infty }\exp\left(\frac{-u^{2}}{2}\right)du.}$$ (2)

Let *X* be Gaussian (*μ*_*X*_,*C*_*X*_) random vector with expected value *μ*_*X*_ and covariance *C*_*X*_ provided that *f*_*X*_(x) is given as:

$$\begin{array}{l} f_{x}(x)=\frac{1}{{\left(2\pi \right)}^{\frac{n}{2}}{\left(\det(x)\right)}^{\frac{1}{2}}}\exp\left(\frac{-1}{2}\left(x-\mu_{x}\right)C_{R}^{-1}\left(x-\mu_{x}\right)\right),\\ \forall \det(C_{x})>0 \end{array}$$ (3)

Suppose the probability function ${\hat{P}}_{j}$, sample variance $\overset{\wedge }{\sigma_{I_{j}}^{2}}$, and indicator function *I*_*j*_ are used for estimating the utility revenues. Let *j* be the number of bins for each histogram evaluation. The above random functions are described as:

$${\hat{P}}_{j}=\frac{1}{N}\sum_{i=1}^{N}I_{j}(R_{i}),$$ (4)

$${\hat{\sigma }}_{1}^{2}=\frac{1}{N-1}{\sum_{i=1}^{N}\left[I_{j}(R_{i})-{\hat{P}}_{j}\right]}^{2},$$

$${\hat{\sigma }}_{I_{j}}^{2}=\frac{n}{N-1}\left[{\hat{P}}_{j}-{\hat{P}}_{j}^{2}\right],\forall I_{j}^{2}(R_{i})=I_{j}(R_{i}),$$

$$\sigma_{{\hat{P}}_{j}}^{2}=\frac{{\hat{\sigma }}_{I_{j}}^{2}}{n}=\frac{1}{N-1}\left[{\hat{P}}_{j}-{\hat{P}}_{j}^{2}\right],$$

$$\in_{R}=\frac{{\hat{\sigma }}_{{\hat{P}}_{j}}}{{\hat{P}}_{j}}=\frac{1}{N-1}\left[1-{\hat{P}}_{j}\right].$$ (5)

In Eq (5), ∈_*R*_ is the relative error of the probability estimate. The term $\sigma_{{\hat{P}}_{j}}^{2}$ will provide the estimated (absolute) error in the probability model.

Let us assume that the data samples *X*_1_,*X*_2_,…,*X*_*n*_ are independent and identically distributed (IID). We assume *m* intervals called histogram bins, which are defined as:

$$\left[e_{j},e_{j+1}\right)|\left(e_{}<\ldots <e_{m+1}\right)∴\left(e_{1}\leq \min X_{i},\max X_{i}\leq e_{m+1}\right).$$ (6)

For preventing any data loss, edge sequence is used when max_*i*_ *X*_*i*_ = *e*_*m*+1_. The interval [*e*_*m*_,*e*_*m*+1_] is preferred in edge sequence estimation. The histogram count for bin *j* is defined as:

$$\begin{array}{l} H_{j}=\sum_{i=1}^{n}\left(I_{\left[e_{j},e_{j+1}\right)}(X_{i})\right)|X_{i}\in \left(e_{i}\leq X_{i}<e_{j+1}\right),\\ \frac{H_{j}}{n}\approx P\left(e_{i}\leq X_{i}<e_{j+1}\right)\approx P\left(X_{i}=j\right). \end{array}$$ (7)

The value of the sample mean *M*_*n*_ converges to the population mean when *n*→∝. The Confidence Interval (CI) is the difference between the random variable and the expected value. The CI is a random set *P*(*m* ∈ [*M*_*n*_−*δ*,*M*_*n*_+*δ*]) = 1−*α*. In probability theory, the term (1−*α*) is called a Confidence Level (CL) and [*M*_*n*_−*δ*,*M*_*n*_+*δ*] is called as CI. The CL is probability for a sample value of the random variable to be present within the CI. In practical applications, we can write *m* = *M*_*n*_ ± *δ* with 100(1−*α*)% probability. In confidence interval, the term *δ* corresponds to $\left(\frac{\sigma y_{\frac{\alpha }{2}}}{\sqrt{n}}\right)$.

#### Theorem

Let *X* be a Gaussian Random Variable (GRV)(*μ*,*σ*). The elements of the set defined as: *X* = {*X*_1_,*X*_2_,*X*_3_,…,*X*_*n*_} are IID. CI estimate of the mean with form (*M*_*n*_(*X*)−*c*) ≤ *μ* ≤ (*M*_*n*_(*X*)+*c*) has a CL $\left(1-\alpha \right),\alpha =Q\left(\frac{c\sqrt{n}}{\sigma }\right)=1-\Phi \left(\frac{c\sqrt{n}}{\sigma }\right)$.

#### Proof

For verifying the above mentioned statement, we consider *X* to be strictly Gaussian. The elements of *X* are exhibiting normal distribution and IID.

#### Definition

A real *R*^*d*^-valued random variable *X* is multi-variant normal or Gaussian if for every vector *t* ∈ *R*^*d*^ the real valued random variable *t*.*X* is normal.

#### Definition

Let *G* be a group with a *σ*-field *F*, such that group operation *x*,*y*→*x*+*y* is a measurable transformation (*G*×*G*,*F*⊗*F*)→(*G*,*F*). Let (Ω,*M*,*P*) be a probability space. A measurable function *X*:(Ω,*M*)→(*G*,*F*) is called a G-valued random variable and the distribution is termed as a probability measure on *G*.

For any constant *c* > 0,

$$\begin{array}{l} :\Leftrightarrow P\left[|M_{n}(X)-\mu_{X}|\geq c\right]\leq \frac{V_{ar}\left[X\right]}{nc^{2}}=\alpha ,\\ :\Leftrightarrow P\left[|M_{n}(X)-\mu_{X}|<c\right]\geq 1-\frac{V_{ar}\left[X\right]}{nc^{2}}=1-\alpha . \end{array}$$ (8)

$$\begin{array}{l} P\left[\left(M_{n}\left(X\right)-c\right)\leq \mu \leq \left(M_{n}\left(X\right)+c\right)\right]\\ =P\left[\left(\mu_{X}-c\right)\leq M_{n}(X)\leq \left(\mu_{X}+c\right)\right]\\ =P\left[-c\leq (M_{n}(X)-\mu_{X})\leq c\right],\\ =P\left[\left(M_{n}\left(X\right)-c\right)\leq \mu \leq \left(M_{n}\left(X\right)+c\right)\right],\\ =P\left[-\frac{c}{\frac{\sigma_{X}}{\sqrt{n}}}\leq \frac{\left(M_{n}(X)-\mu_{X}\right)}{\frac{\sigma_{X}}{\sqrt{n}}}\leq \frac{c}{\frac{\sigma_{X}}{\sqrt{n}}}\right],\\ =1-2Q\left(\frac{c\sqrt{n}}{\sigma_{X}}\right)=\left(1-\alpha \right). \end{array}$$ (9)

The stochastic random demand *D*(*t*) is categorized as: (a) fixed demand *D*_*F*_(*t*) and (b) variable demand *D*_*V*_(*t*). Fixed demand is the energy consumption of the fixed loads, such as lights, bulbs, and constant energy consumption loads. Variable demand is the energy consumption of the variable loads, such as washing machine, electric car charging, and electric cooling system. The utility revenue *R* is the random function of consumer’s demand *D* and price *p* of electricity. The total demand model is summarized as:

$$\begin{array}{l} :\Leftrightarrow D_{F}\left(t\right)=\left[\sum_{i=1}^{n}\left(D_{F_{t}}(t),\ldots ,D_{F_{n}}(t)\right),\forall D_{F}(t)>0\right],\\ D_{F_{\min}}\left(t\right)\leq D_{F}\left(t\right)\leq D_{F_{\max}}\left(t\right),\\ D_{v}\left(t\right)=\sum_{i=1}^{n}\left(D_{v_{t}}(t),\ldots ,D_{v_{n}}(t)\right),\forall D_{v}(t)>0,\\ D_{v_{\min}}\left(t\right)\leq D_{v}\left(t\right)\leq D_{v_{\max}}\left(t\right). \end{array}$$ (10)

The total revenue function *R*(*t*) is classified as: (a) fixed revenue *R*_*F*_(*t*) and (b) variable revenue *R*_*V*_(*t*). Fixed revenue model, variable revenue model, and revenue constraints are described as:

$$\begin{array}{l} :\Leftrightarrow R=f_{R}\left(D,P\right),\\ D_{F}\left(t\right)\in R_{f}\left(t\right),D_{v}\left(t\right)\in R_{v}\left(t\right),\\ R\left(t\right)=R_{F}\left(t\right)+R_{v}\left(t\right). \end{array}$$ (11)

$$\begin{array}{l} :\Leftrightarrow R_{F}\left(t\right)=\left[\sum_{i=0}^{n}P\left(\int_{0}^{d_{i+1}}\partial D_{F}\right),\forall R_{F}\left(t\right)>0\right],\\ :\Leftrightarrow R_{v}\left(t\right)=\left[\sum_{i=0}^{n}P\left(\int_{0}^{d_{i+1}}\partial D_{v}\right),\forall R_{v}\left(t\right)>0\right],\\ R_{F}\left(t\right)<R_{v}(t),\\ R_{F\min}\left(t\right)\leq R_{F}\left(t\right)\leq R_{F\max}\left(t\right),\\ R_{v\min}\left(t\right)\leq R_{v}\left(t\right)\leq R_{v\max}\left(t\right). \end{array}$$ (12)

The above mentioned stochastic model is modelled as MVGDF. The consumer demands are time-dependent random functions. The time-varying energy consumption is the sample outcome of the random events. In SG, consumer demands vary as a GDF and the outcomes of the Gaussian Event (GE) will also be a Gaussian.

## 4. Simulation and Results

### 4.1 Stochastic Analysis

To validate the MVGDF, consumer demands data are collected from the local grid station [29],[30]. The data set included consumer demands, weather parameters, such as temperature, humidity, precipitation, and generation capacity of the utility. The consumer demand is taken as a random input and outcomes of the numerical simulations are: (a) fixed revenue *R*_*F*_(*t*) and (b) variable revenue *R*_*v*_(*t*), probabilities of *R*_*F*_(*t*) and *R*_*v*_(*t*), and Relative Errors (REs) in the proposed models. The random inputs of the data samples, such as 10^7^, 10^8^, and 10^9^ samples obeys GDF characteristics. Utility revenues, probability estimates, and REs are evaluated in each set of data sample. For optimized utility revenues, SMC simulations are performed using MATLAB. Moreover, complete statistics of MVGDF *R*_*F*_(*t*) and *R*_*v*_(*t*) is elaborated using statistical analysis of the outcomes from the numerical simulations. Furthermore, Confidence Intervals (CIs) and REs in the aforementioned stochastic model is also evaluated for various data samples.

**Fig 2:**
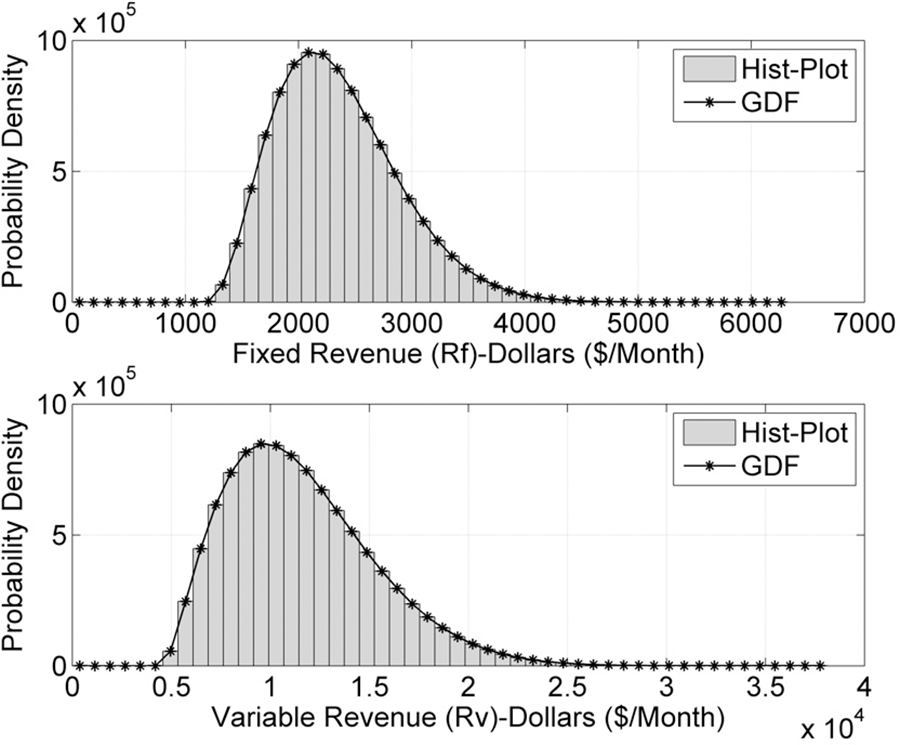
*R*_*F*_(*t*) and *R*_*v*_(*t*) for *n* = 10^7^ samples.

**Fig 3:**
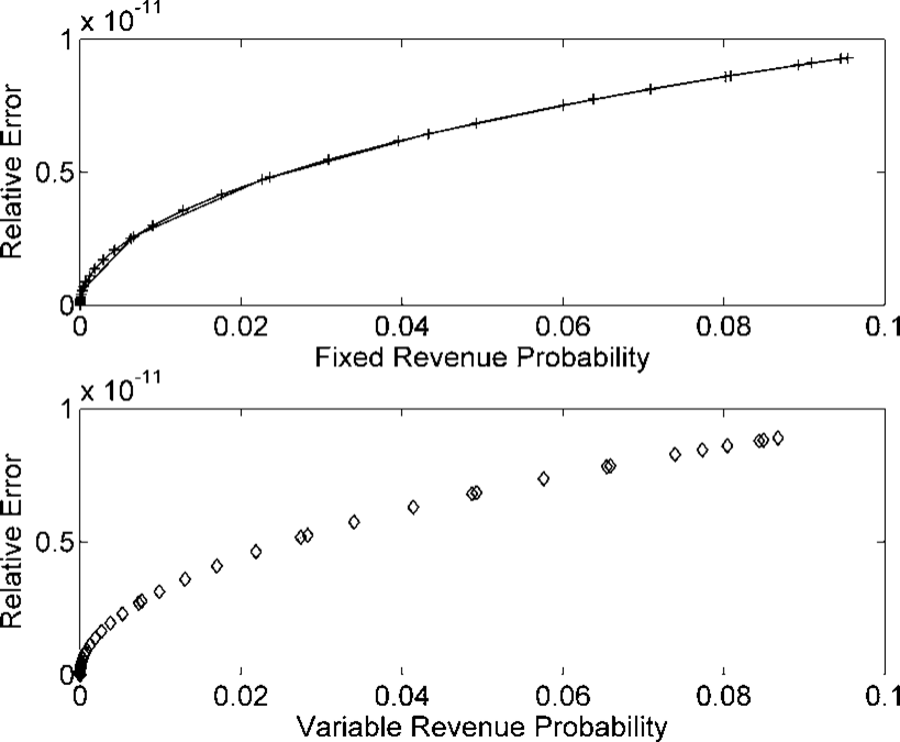
REs in *R*_*F*_(*t*) and *R*_*v*_(*t*) probabilities for *n* = 10^7^ samples.

**Fig 4:**
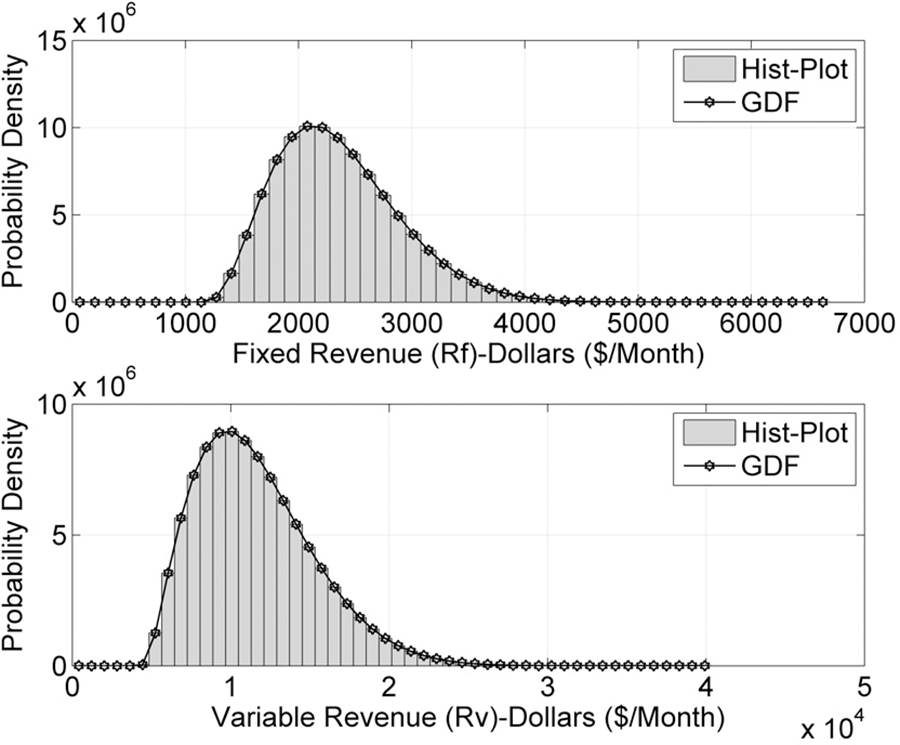
*R*_*F*_(*t*) and *R*_*v*_(*t*) for *n* = 10^8^ samples.

**Fig 5:**
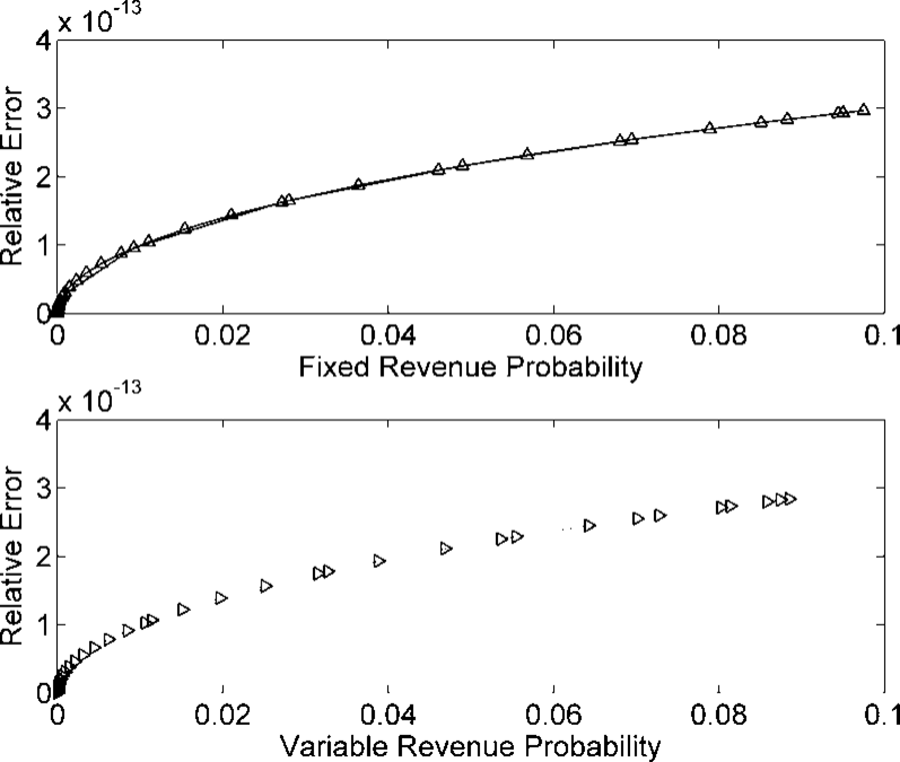
REs in *R*_*F*_(*t*) and *R*_*v*_(*t*) for *n* = 10^8^ samples.

Figs 2 and 3 describe the numerical simulations outcomes for input data samples *n* = 10^7^. Fig 2 presents *R*_*F*_(*t*) and *R*_*v*_(*t*) plot using histograms and GDF. Fig 3 indicate the REs in the estimated model for *n* = 10^7^ data samples. Figs 4 and 5 describe the probability models of *R*_*F*_(*t*) and *R*_*v*_(*t*) for *n* = 10^8^ data samples. The probability density function increases for *R*_*F*_(*t*) and *R*_*v*_(*t*), compared to the 10^7^ data samples cases.

**Fig 6:**
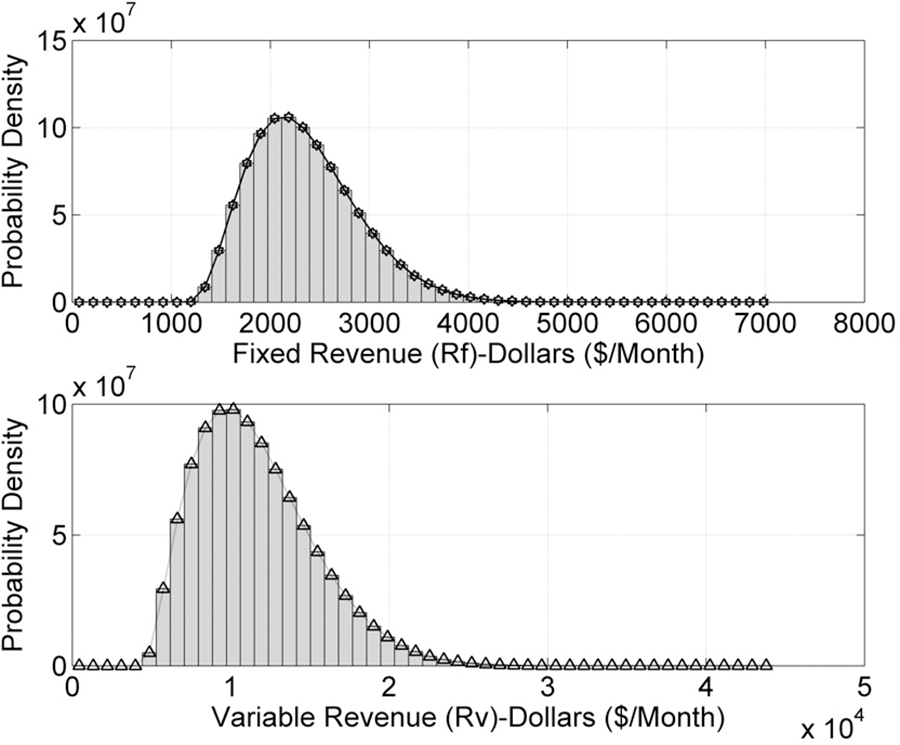
*R*_*F*_(*t*) and *R*_*v*_(*t*) probabilities for *n* = 10^9^ samples/

**Fig 7:**
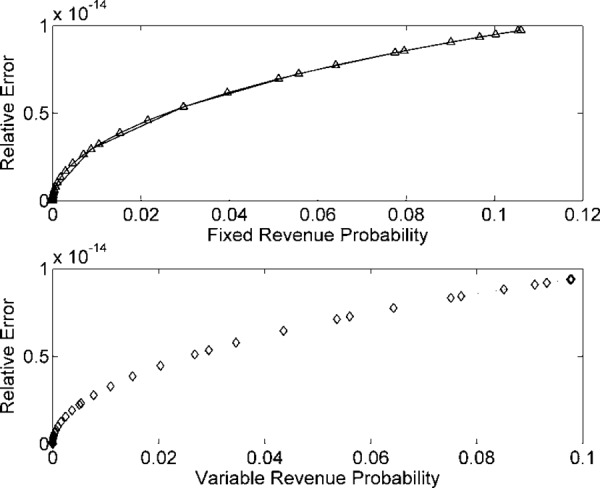
REs in *R*_*F*_(*t*) and *R*_*v*_(*t*) for *n* = 10^9^ samples.

Figs 6 and 7 are the estimates for *n* = 10^9^ data samples of energy demand. REs are reduced and probability factor of *R*_*F*_(*t*) and *R*_*v*_(*t*) has increased further. Figs 6 and 7 reflect higher probability densities and minimum REs for *n* = 10^9^ random samples. Fig 7 indicate the least RE and highest density in the probability estimate, compare to above models. From probability theory, the REs will approach zero when input data samples are taken close to infinity. The factor of accuracy in the probability estimates also increases likewise.

**Fig 8:**
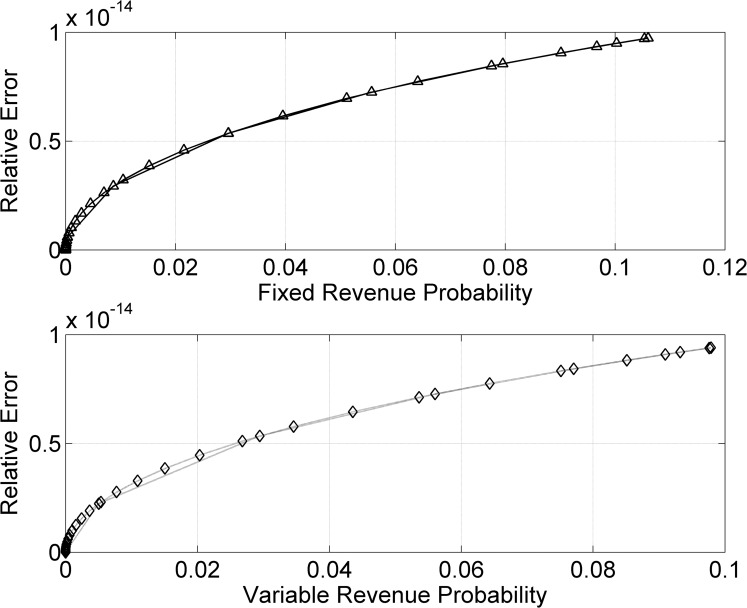
G-Matrix Plot for the MVGDFs with fixed load as a predictor for *n* = 10^7^ data samples.

**Fig 9:**
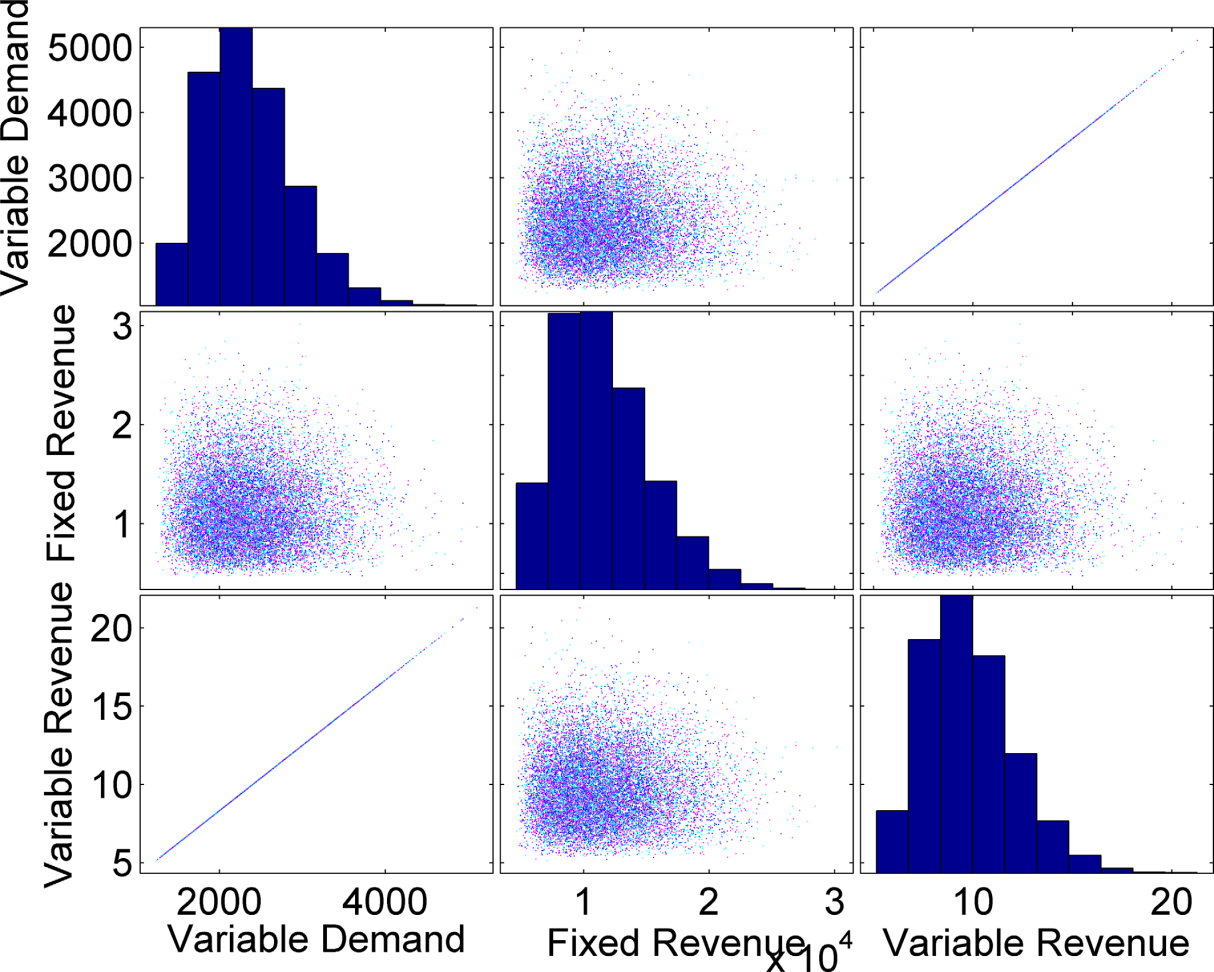
G-Matrix Plot for the MVGDFs with variable load as a predictor for *n* = 10^7^ data samples.

Figs 8 and 9 elaborate the analysis of multi-variant data using two-dimensional matrix scatter plots. The matrix scatter plots are three outcomes generated from one predictor variable. In Fig 8, fixed load is considered as a predictor, while variable load is the predictor in Fig 9. The above mentioned plots also describe the multi-correlations among dependent and independent variables of the MVGDFs. A Univariate histogram is presented for each variable relationship.

### 4.2 Statistical Analysis

Table 1 presents the monthly utility load consumptions of the local grid station [29], [30],. The Fixed Load (FL) and Variable Load (VL) consumptions are described for residential, commercial, and industrial loads. The energy consumption of each type of load varies, for example, industrial loads have highest energy consumption than commercial and residential loads. Table 2 discusses the Utility Load Curve Periods (ULCPs) during off-peak period, mid-peak period, and peak periods. The energy consumption during peak-period is high than mid-peak periods and off-peak periods. Table 3 and Table 4 elaborate the statistics of *R*_*F*_(*t*) and *R*_*v*_(*t*) for various consumer demand samples. Statistical analysis include parameters, such as maximum (max) value, minimum (min) value, Standard Deviation (SD), Variance (V), Co-Variance (CV), and CIs. The CIs values are calculated on 99% probability. Table 5 and Table 6 describe the statistics of REs in *R*_*F*_(*t*) and *R*_*v*_(*t*) in above mentioned models for various data samples. Moreover, REs approaches to a minimum value for *n =* 10^9^ samples. Furthermore, the mean value of RE in the estimated values reaches a very low value in the order 10^−15^. Finally, the variation of the samples in the estimated values is approximately negligible. This factor ensures the high reliability of the estimated values.

**Table 1:** Consumers Monthly Utility Load Consumptions.

Load-MW	FL	VL
Residential	1 MW—2 MW	3 MW—7 MW
Commercial	2 MW - 4MW	5 MW - 15MW
Industrial	2 MW—5 MW	5 MW—20 MW

**Table 2:** Utility Load Curve Analysis.

ULCP	Duration	Tariff-FL	Tariff -VL
Off-Peak period	0.00 AM—7.00 AM	Rs. 8.00	Rs. 8.00
Mid-Peak period	8.00 AM—15.00 AM	Rs. 8.00	Rs. 10.00
Peak Period	16.00 AM -23.00 AM	Rs. 8.00	Rs. 12.00

**Table 3:** Statistical Analysis of *R*_*f*._

Samples-*n*	Max	Min	Mean	Median	STD	V	(CI)exp3	CV
10^7^	6.132e3	0	2.349e3	2.281e3	541.51	2.932e5	[2.349, 2.348]	2.932e5
10^8^	6.849e3	0	2.348e3	2.281e3	541.35	2.930e5	[2.349, 2.348]	2.930e5
10^9^	7.052e3	0	2.348e3	2.281e3	541.325	2.93e5	[2.349, 2.348]	2.93e5

**Table 4:** Statistical Analysis of *R*_*v*._

Samples-*n*	Max	Min	Mean	Median	STD	V	(CI)exp4	CV
10^7^	3.936e4	0	1.158e4	1.104e4	3.734e3	1.394e7	[11.58,11.58]	1.394e7
10^8^	4.148e4	0	1.158e4	1.104e4	3.735e3	1.395e7	[11.58, 11.58]	1.395e7
10^9^	4.423e4	0	1.158e4	1.104e4	3.734e3	1.394e7	[11.58, 11.58]	1.394e7

**Table 5:** Statistical Analysis of Relative Errors in *R*_*f*_ estimation.

Samples-*n*	Max	Min	Mean	STD	V
10^7^	9.171e-12	0	2.8e-12	3.324e-12	1.105e-23
10^8^	3.046e-13	0	8.355e-14	1.085e-13	1.177e-26
10^9^	9.735e-15	0	2.602e-15	3.455e-15	1.193e-29

**Table 6:** Statistical Analysis of Relative Errors in *R*_*v*_ estimation.

Samples-*n*	Max	Min	Mean	STD	V
10^7^	8.948e-12	0	2.878e-12	3.266e-12	1.066e-23
10^8^	2.893e-13	0	8.861e-14	1.050e-13	1.104e-26
10^9^	9.397e-15	0	2.708e-15	3.388e-15	1.148e-29

From the above mentioned statistical analysis and SMC simulations, present and future estimates of the SG can be calculated and predicted. The important SG parameters for estimation are listed as:

- Demand-supply management;
- Present and future energy demands based on the past population estimates;
- Generation units expansion;
- Energy costs estimations, such as fuel costs;
- Present and future transmission-line losses estimations based on past observations;
- Installment of Distributed Generators (DGs) with the SG system;
- Statistical weather data incorporation for predicting power system controlling parameters, such as power-flows; and
- Utility financial and economic development plans.

The stochastic analysis can be utilized for planning, re-shaping, and modifying overall utility characteristics. With such demand and economic estimates, electrical utility will be able to take further steps for predicting various other electrical parameters, such as voltage instability and transmission-line losses. Moreover, the probabilistic estimates will help electrical utility while bidding for demand response programs in deregulated energy market of the SG. Furthermore, consumer’s satisfaction will be increased and quality-of-service will be upgraded. Finally, several other predictive models of the SG can be estimated using above methodology.

### 4.3 Weather Data Analysis Using Correlation and Regression Schemes

#### 4.3.1 Correlation Schemes

**Fig 10:**
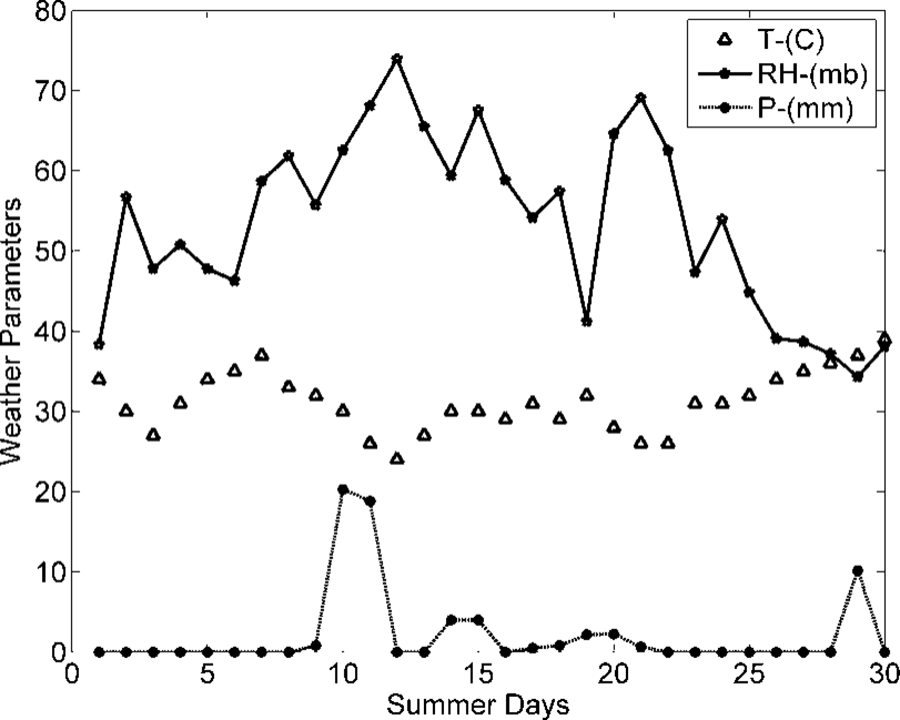
Variation in Weather Data Parameters.

In this section, weather parameters are correlated with each other using correlation schemes, such as Pearson (P), Spearman (S), and Kendall (K). The summer weather parameters taken into consideration are Temperature *T*, Humidity *H*, and Precipitation *P* recorded from the local grid station of Pakistan, as shown in Fig 10 [31].

**Fig 11:**
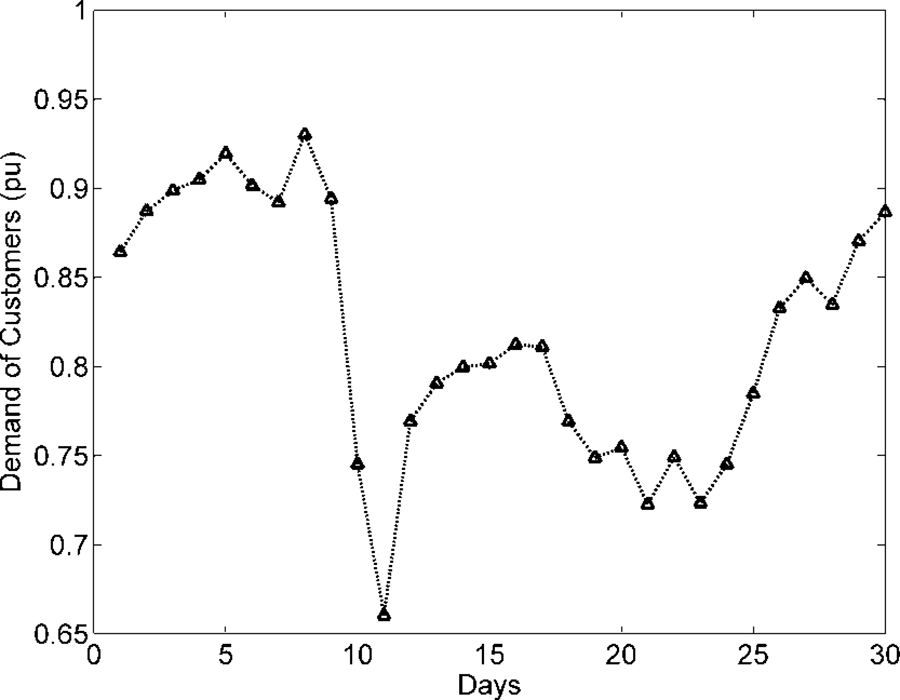
Variation in Monthly Consumers Load pu (Summer).

**Fig 12:**
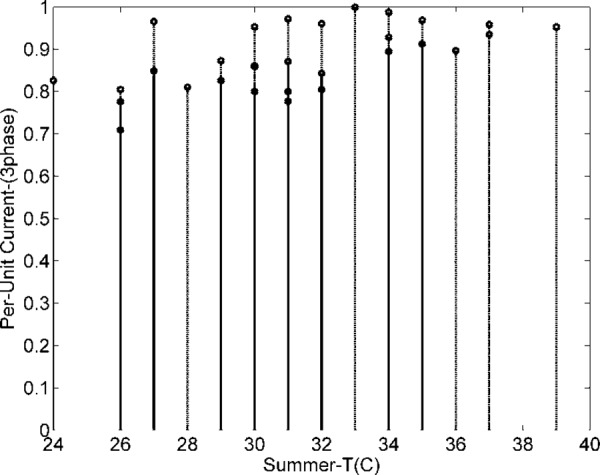
Variation in Consumers Demand with *T*.

**Fig 13:**
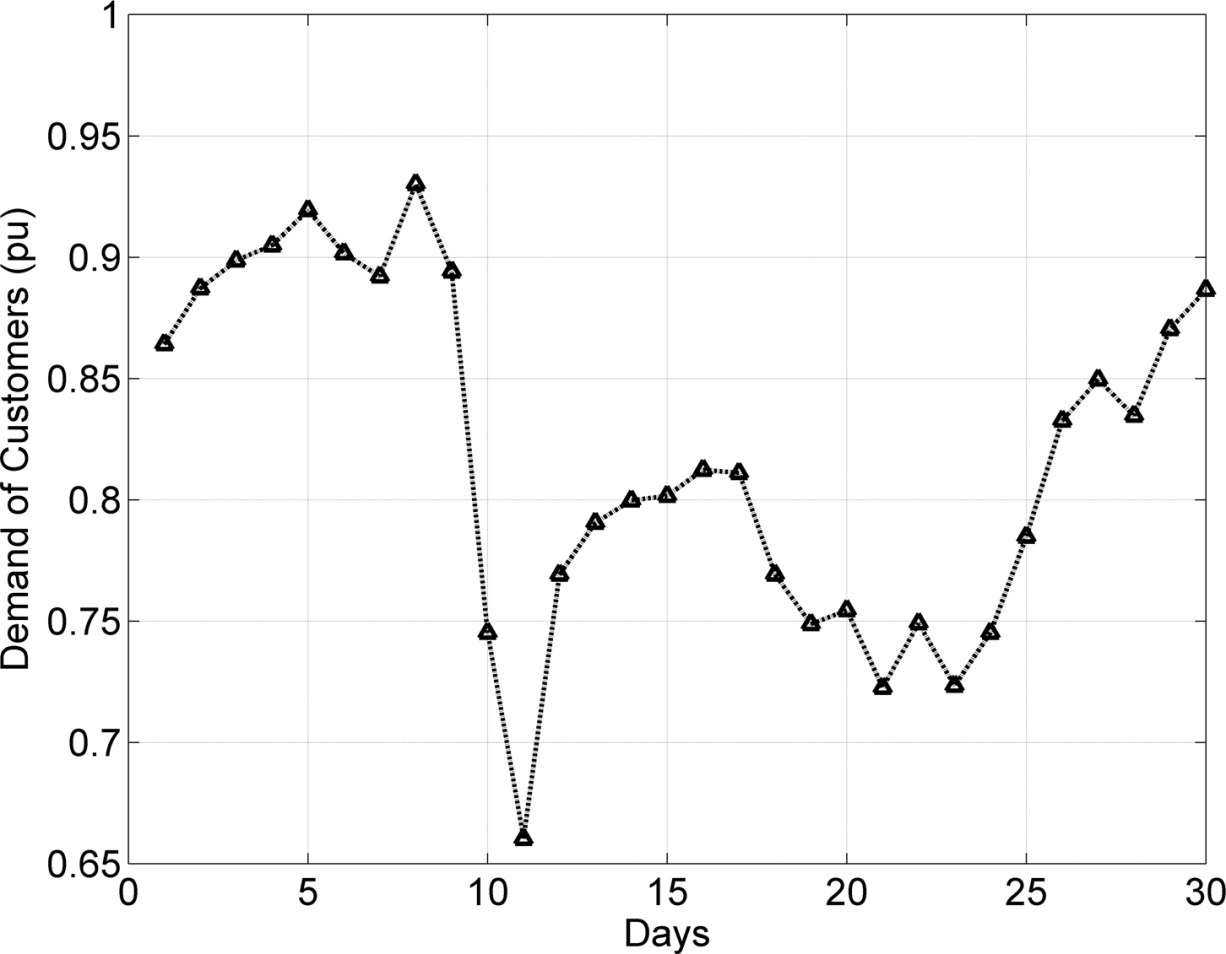
Variation in Consumers Current Demand with *T*.

**Fig 14:**
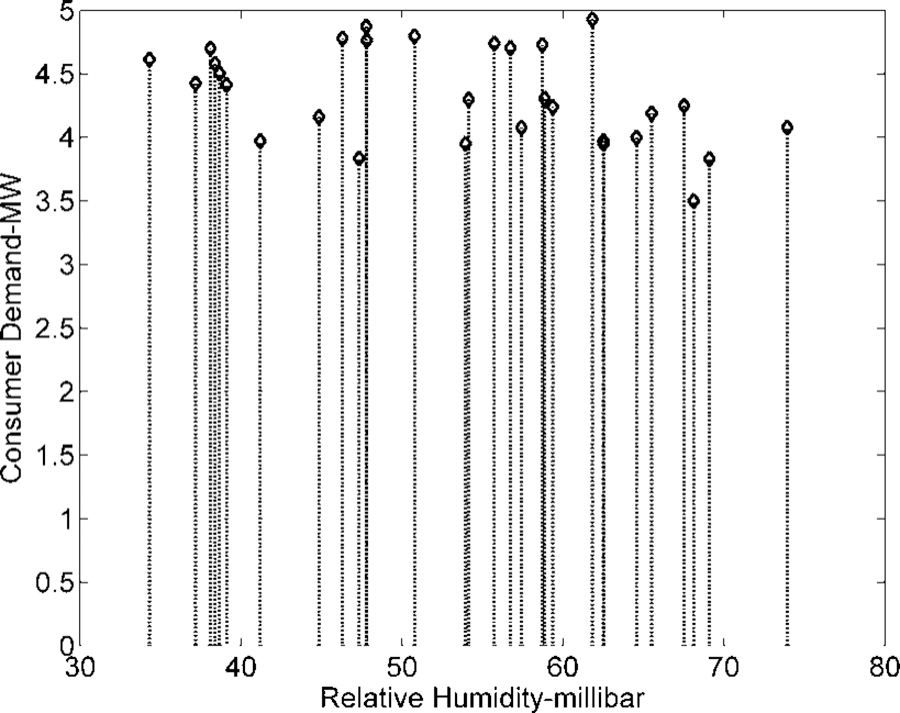
Variation in Consumers Demand with *H*.

**Fig 15:**
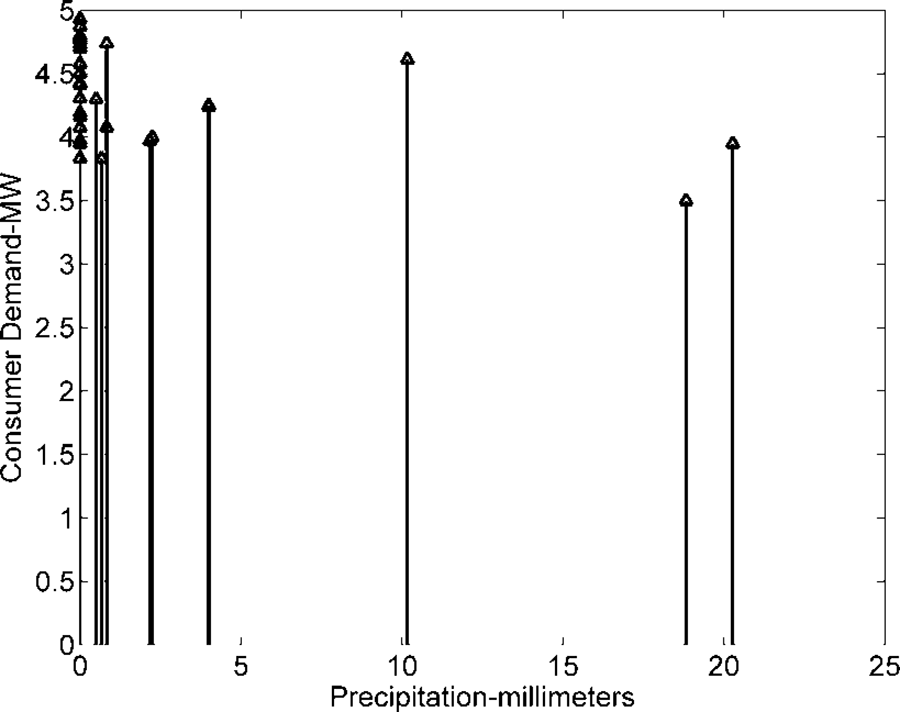
Variation in Consumers Demand with *P*.

The demand of consumers (power demand) *L* is a dependent variable, while weather data variables are independent. The objective is to investigate and analyze correlations between dependent and independent variables (*T*, *H*, and *P*). Dependent variable (load) is graphically analyzed in Fig 11. The dependencies of power and current (pu) demand with respect to *T* are shown in Figs 12 and 13. Similarly, consumer power demand variations with respect to *H* and *P* are presented in Figs 14 and 15.

From aforesaid graphical analysis, consumer demands are highly dependent on weather parameters, which directly affect the lifestyles of the people. Among various weather parameters, *T* and *H* effects the most than P as compared to consumer demands. Moreover, current increase on transmission lines is obvious due to the increase in *T*, which assures that weather forecasting directly relates to the consumers demand in smart grid. Furthermore, without forecasting generation capacity, load growth, losses calculations, and optimal revenue estimation will not be astimated optimally.

In the following tables namely, Table 7, Table 8, Table 9 and Table 10, inter-relationships are explored using P, S, and K schemes. The terms V1, V2, V3, and V4 symbolize Load *L*, Temperature *T*, Humidity *H*, and Precipitation *P*. In Table 7, the effect of *L* on *T*, *H*, and *P* is investigated using aforesaid schemes. The correlation of V1 with respect to V2, V3, and V4 is presented using *R-software*. The positive correlations indicate strong association of *L* with *T*, *H*, and *P*, while negative quantities indicate weak relationships among variable. For example, correlation of V1 with V2 is 60.22% with P scheme, while 56.78% and 40.82% with S and K schemes. Similarly, there is a weak association of V1 with V2 and V3. Following above mentioned methodology, correlations of *T* with *L*, *H*, and *P* is presented in Table 8. Table 9 and Table 10 discuss the effects of *H* and *P* on dependent and independent variables. The relationship of *L* and *T* bear a very strong association with *H* and *P*. This shows that critical investigation of the parameters will play foremost role in analyzing smart grid systems, load forecasting, demand predictions, and energy estimations. Moreover, among these weather parameters, temperature performs a significant role in analyzing smart dynamical energy systems. Furthermore, in probabilistic utility revenue estimations, load and temperature data must be used for revenue predictions, energy estimations, and demand-supply management.

**Table 7:** Load (*L*) Correlation WRT Temperature (*T*), Humidity (*H*), and Precipitation (*P*).

*L*-Correlation	P-Correlation Value (*T*, *H*, *P*)	S-Correlation Value (*T*, *H*, *P*)	K-Correlation Value (*T*, *H*, *P*)
V1~V2	0.6022	0.5678	0.4082
V1~V3	-0.4296	-0.4096	-0.2880
V1~V4	-0.4164	-0.4213	-0.3265

**Table 8:** *T* Correlation WRT *L*, *H*, and *P*.

*T*-Correlation	P-Correlation Value (*L*, *H*, *P*)	S-Correlation Value (*L*, *H*, *P*)	K-Correlation Value (*L*, *H*, *P*)
V2~V1	0.6022	0.5678	0.4082
V2~V3	-0.7733	-0.8023	-0.6630
V2~V4	-0.1487	-0.2458	-0.1952

**Table 9:** *H* Correlation WRT *L*, *T*, and *P*.

*H*-Correlation	P-Correlation Value (*L*, *T*, *P*)	S-Correlation Value (*L*, *T*, *P*)	K-Correlation Value (*L*, *T*, *P*)
V3~V1	-0.4296	-0.4096	-0.2880
V3~V2	-0.7733	-0.8023	-0.6630
V3~V4	0.2168	0.3253	0.2606

**Table 10:** *P* Correlation WRT *L*, *T*, and *H*.

*P*-Correlation	P-Correlation Value (*L*, *T*, *H*)	S-Correlation Value (*L*,*T*,*H*)	K-Correlation Value (*L*, *T*, *H*)
V4~V1	-0.4164	-0.4213	-0.3265
V4~V2	-0.1487	-0.2458	-0.1952
V4~V3	0.2168	0.3253	0.2606

#### 4.3.2 Multi-Linear Regression System Model

The model for multi-linear regression is described in Eq (13) and Eq (14). The dependent and independent parameters are analyzed using *R-platform*. This investigation is based on conditional probability distribution of load with respect to weather data parameters. The term *Y* indicates the dependent variable (load), *X* is independent weather data parameters, and *ε* is the error variable. *β* is the regression coefficient, while B_o_ is the intercept. Using aforementioned model, three varying weather data parameters are analyzed for association with load. In Table 11, the Load L parameter (V1) is varied as 2V1, 0.5 V1 and V1.

$$\begin{array}{l} y_{i}=\beta_{1}x_{i1}+\beta_{2}x_{i2}+\ldots +\varepsilon_{i},\\ i=1,2,\ldots ,n,\\ Y=X\beta +\varepsilon ,\\ Y=\left(\begin{array}{c} y_{1}\\ y_{2}\\ .\\ .\\ .\\ y_{n} \end{array}\right),X=\left(\begin{array}{c} x_{1}^{T}\\ x_{2}^{T}\\ .\\ .\\ .\\ x_{n}^{T} \end{array}\right),\beta =\left(\begin{array}{c} \beta_{1}\\ \beta_{2}\\ .\\ .\\ .\\ \beta_{n} \end{array}\right),\varepsilon =\left(\begin{array}{c} \varepsilon_{1}\\ \varepsilon_{2}\\ .\\ .\\ .\\ \varepsilon_{n} \end{array}\right). \end{array}$$ (13)

$$\begin{array}{l} Y=B_{0}+B_{1}X_{1}+B_{2}X_{2}+\ldots +B_{k}X_{k},\\ k=1,2,\ldots ,n. \end{array}$$ (14)

**Table 11:** Simple-Linear Regression Analysis of Load.

Load-*L*	Model	*R*^*2*^	*R*
V1-2V2-V3-V4	*L* = 3.817+0.06931V2+0.01206V3+(-0.05125)V4	0.4251	65.19%
0.5V1-V2-2V3-V4	*L* = 3.8127+0.1386V2+0.006029V3+(-0.05125)V4	0.4251	65.19%
2V1-2V2-0.5V3-0.5V4	*L* = 3.8172+0.06931V2+0.02412V3+(-0.1025)V4	0.4251	65.19%

**Fig 16:**
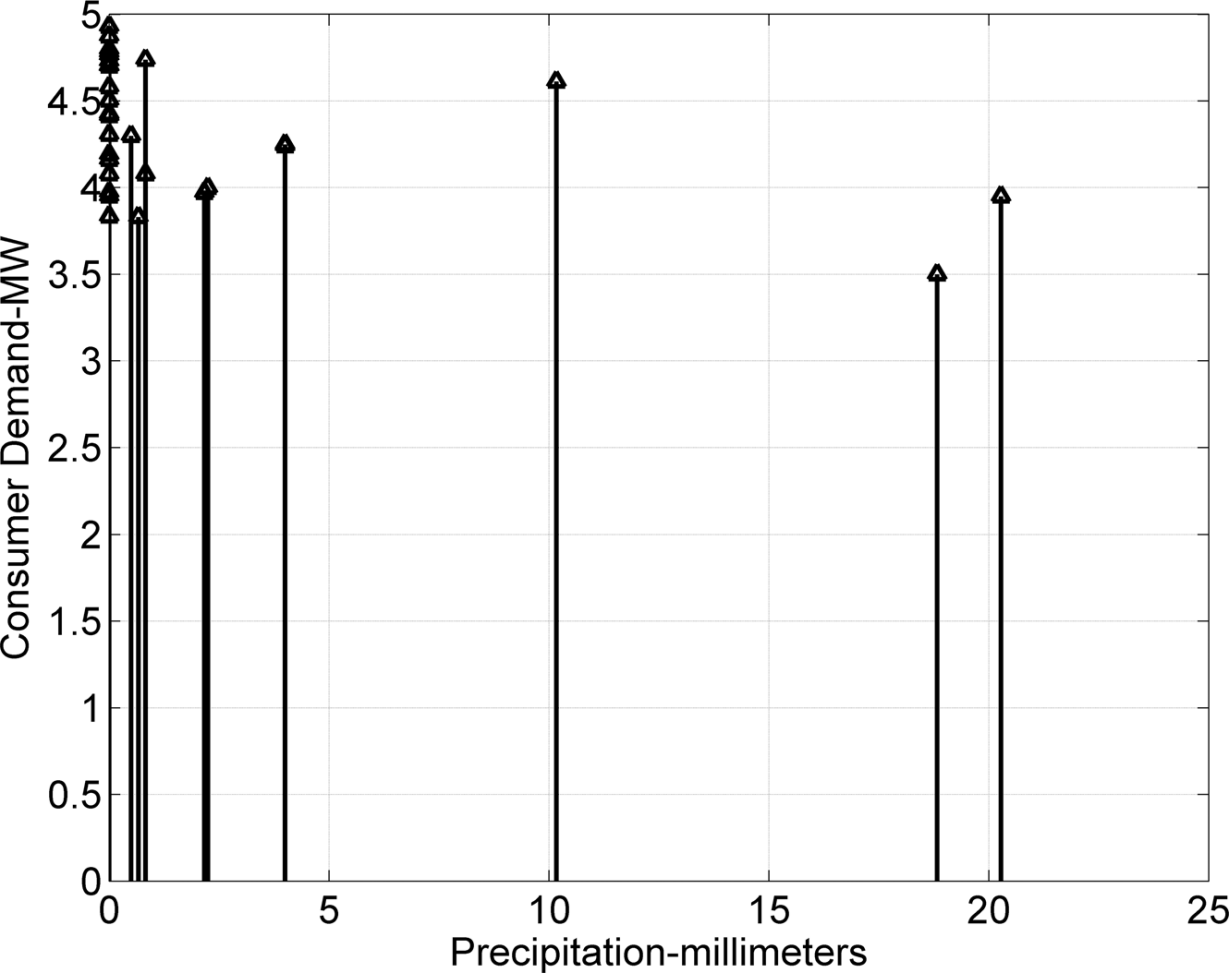
Multi-Linear Regression Analysis.

The relation of this varying load is analyzed with respect to varying weather data or predictor variables, which are Temperature *T* (V2), Humidity *H* (V3), and Precipitation *P* (V4). This shows that in each case there exists a strong association of load with varying weather data parameters. This association is 65.19%, which indicates a strong evidence of load forecasting or demand forecasting in smart grid networks using weather data parameters. The multi-regression scatter plots are described in Fig 16.

Various test cases of varying dependent and independent variable are analyzed and plotted in R. We see that a very strong association, such as R = 1.0 and R = 60% exists for dependent variable with independent variables. However, there is a weak relation for humidity and precipitation with other dependent and independent parameters. We conclude that stability and steady-state performance is the foremost feature for smart grids. The role of stochastic models and load prediction and estimations are evident from above critical analysis. The weather data parameters influence the living standards of consumers, which in turn effect the demand of electric supply. This shows that probabilistic evaluation and statistical analysis of consumer demands plays a pivotal role in smart grid load monitoring, management, prediction, stability, and control. Moreover, economic and financial developments can be forecasted and predicted using above analysis.

## 5. Conclusions and Future Work

Time-varying processes are stochastic in nature, such as consumer’s demand in smart grid environment. The probability estimation of the uncertain events is fairer when random samples are close to infinity. We elaborated probabilistic utility revenue functions using GDF and comprehensive statistical analysis. The energy demands of the consumers are modeled as time-variant and random input functions. The probability estimation presented a demand-revenue model for consumer energy demand forecasting and utility revenue estimates. The correlations and regression analysis of weather data analysis is also performed using local grid data and weather data. We concluded that the present and future estimates of utility revenues are highly dependent on the time-varying consumer demands and weather data.

The effect of consumer’s demand on the utility revenues can be analyzed in various domains of stochastic processes. The utility stochastic analysis can be extended considering all random input variables, such as price of electricity and consumer participation in demand response programs. The MVGDF and probabilistic demand-revenue models can be further evaluated using advanced MC and multi-canonical MC simulations. The aforementioned models can also be described within various inter-connected areas of SG, such as local area SG system and wide area SG system. In near future, load forecasting will be completed using various prediction schemes for short-term and long-term forecasting.
